# Endogenous retroviruses and response to immune checkpoint inhibitors: mechanisms, clinical evidence, and therapeutic implications

**DOI:** 10.3389/fimmu.2026.1835325

**Published:** 2026-05-20

**Authors:** Fanyuan Wu, Quezhu Danzeng, Runxi Wu, Yi Shen, Guang Shi

**Affiliations:** 1 Department of Hematology and Oncology, The Second Hospital of Jilin University, Changchun, China; 2 Department of Spinal Surgery, The Second Hospital of Jilin University, Changchun, China; 3 Department of Nuclear Medicine, China-Japan Union Hospital of Jilin University, Changchun, China

**Keywords:** endogenous retroviruses, epigenetic regulation, immune checkpoint inhibitors, tumor microenvironment, viral mimicry

## Abstract

Endogenous retroviruses (ERVs) are epigenetically silenced remnants of ancient retroviral integrations that comprise ~8% of the human genome. In cancer, DNA hypomethylation and chromatin remodeling—spontaneously or induced by epigenetic therapies—can derepress ERV loci, leading to abundant ERV-derived double-stranded RNA (dsRNA) and, in some cases, immunogenic ERV proteins. Accumulated dsRNA is primarily sensed by MDA5/RIG-I and TLR3, activating MAVS/TRIF signaling to induce IRF3/7- and NF-κB–dependent type I interferons and interferon-stimulated genes. This viral mimicry enhances antigen processing and MHC-I presentation, recruits CXCR3+ effector lymphocytes via CXCL9/10/11, promotes dendritic-cell activation, reduces immunosuppressive populations, and can convert immune-cold tumors into immune-active states while also increasing PD-L1 expression. Clinical evidence from retrospective cohorts and early prospective studies supports ERV signatures as biomarkers for immune checkpoint inhibitor (ICI) response, often independent of PD-L1 or tumor mutational burden, and enables ERV-based stratification. Therapeutic strategies that induce ERVs or target ERV antigens may sensitize tumors to ICIs, although assay standardization, prospective validation, and long-term safety remain key challenges.

## 1 Introduction

Endogenous retroviruses (ERVs) are remnants of ancient exogenous retroviruses that integrated into the host germline genome, accounting for about 8% of the human genome (1–3). In humans, these elements are primarily known as human endogenous retroviruses (HERVs), which are vertically inherited, retrovirus-derived sequences rather than latent infectious retroviruses. Having originated from germline infections and subsequently become fixed as proviral integrations, HERV sequences are present in virtually all nucleated cells as stable components of the human genome. More broadly, retroelements constitute a substantial proportion of the human genome, whereas ERV-derived sequences include both relatively intact proviral elements and highly degenerated fragments (4, 5).

A canonical HERV locus exhibits the structure of an integrated provirus, typically flanked by two long terminal repeats (LTRs) and containing internal retroviral genes, including gag, pro, pol, and env (6). However, most HERV insertions have undergone extensive evolutionary degeneration through point mutations, insertions, deletions, recombination events and homologous recombination between the two LTRs (7). Consequently, HERVs exist in the human genome in multiple forms, including full-length or near full-length proviruses, defective proviral remnants, truncated internal fragments, mosaic or recombinant elements and solo LTRs, the latter representing the most abundant HERV-derived traces (8, 9).

Based on sequence similarity and phylogenetic relationships with exogenous retroviruses, HERVs are commonly categorized into three classes. Class I includes gammaretrovirus- and epsilonretrovirus-like elements, such as HERV-H, HERV-W, HERV-E, HERV-T, and HERV-I. Class II consists of betaretrovirus-like elements, mainly represented by the HERV-K/HML groups. Class III encompasses spumavirus-like elements, such as HERV-L and HERV-S (10). Consistent with this classification, Vargiu et al. identified 3173 relatively complete HERV proviral sequences in the human genome assembly GRCh37/hg19, of which 1214 canonical sequences were assigned to 39 canonical HERV clades, while many noncanonical elements showed mosaic structures generated by recombination or secondary integrations (11). Although no replication-competent HERV has been definitively identified in the human genome, several loci retain open reading frames or coding potential, particularly the relatively recent HERV-K (HML-2) elements (12). Additionally, some HERV-derived envelope genes have been domesticated for physiological functions, such as syncytin-1 from HERV-W and syncytin-2 from HERV-FRD (11).

**Figure 1:**
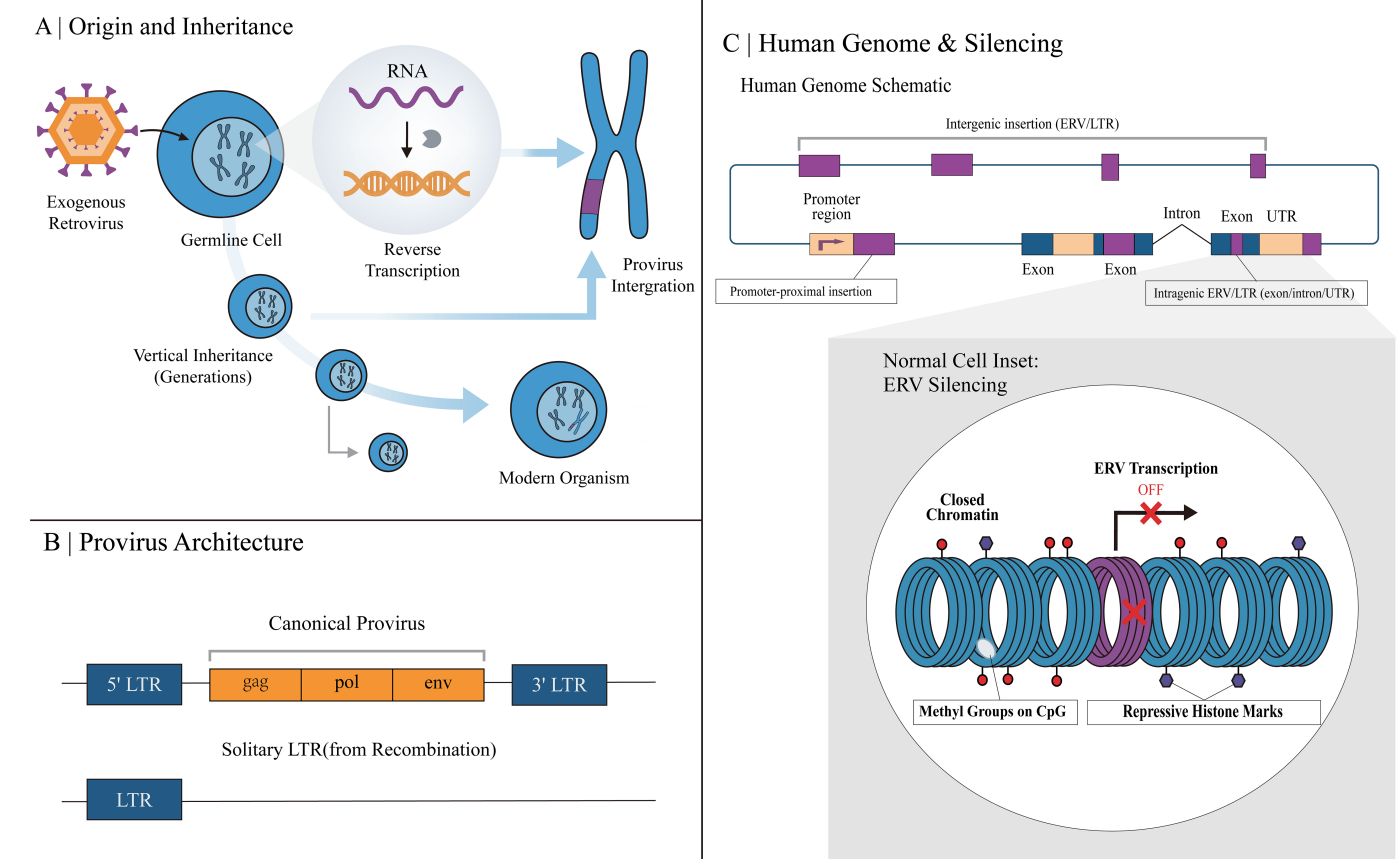
Evolutionary origin, genomic organization, distribution, and physiological silencing of endogenous retroviruses (ERVs). **(A)** ERVs originate from ancient exogenous retroviruses that infected germline cells. Following reverse transcription of the viral RNA genome into DNA, the viral sequence became integrated into the host chromosome as a provirus. Once fixed in the germline, the integrated retroviral sequence was vertically transmitted to offspring in a Mendelian manner and accumulated across generations in modern genomes. **(B)** Canonical ERVs retain the typical proviral structure LTR-gag-pol-env-LTR, in which the long terminal repeats (LTRs) flank the retroviral coding regions. In humans, however, most ERV loci are no longer replication-competent because they have accumulated mutations, internal deletions, or rearrangements. Homologous recombination between the two LTRs can remove the internal coding region and generate a solitary LTR. **(C)** ERV-derived sequences are widely but unevenly distributed throughout the human genome, occurring in intergenic regions, promoter-proximal regions, and intragenic regions such as exons, introns, and untranslated regions (UTRs). Depending on their genomic position, ERVs or solitary LTRs may influence nearby gene expression by serving as regulatory elements, including alternative promoters or enhancers. The inset illustrates ERV silencing in normal somatic cells, where DNA methylation at CpG sites and repressive histone modifications, including H3K9me3 and H3K27me3, promote chromatin compaction and transcriptional repression, thereby preventing aberrant ERV expression, maintaining genomic stability, and limiting inappropriate innate immune activation.

In healthy tissues and under normal physiological conditions, the majority of ERVs are tightly suppressed by epigenetic mechanisms, thereby preserving genomic stability. This state of silencing carries profound immunological significance. On one hand, it suppresses the expression of potentially immunogenic self-antigens, such as proteins and nucleic acids derived from ERVs, preventing the immune system from mounting inappropriate responses against endogenous genomic elements (13, 14). On the other hand, even in their silenced state, ERVs exhibit a controlled, low level of expression; this sustained expression provides the immune system with a low-level, sustained exposure to signals resembling pathogen-associated molecular patterns (PAMPs). Consequently, it contributes to maintaining baseline activity of the interferon pathway and a state of antiviral vigilance in the organism, typically without eliciting overt inflammatory responses (**Figure 1**) (13, 15, 16).

Under pathological conditions such as cancer or HIV infection, malignant transformation, viral infection, or epigenetic disruption can lead to aberrant reactivation of ERVs, whose products trigger immune responses (17). The most studied mechanism involves double-stranded RNA (dsRNA) transcribed from ERVs, which is detected by intracellular pattern recognition receptors and activates the type I interferon pathway, eliciting antiviral and antitumor responses (18, 19). Proteins encoded by ERVs, normally silent in healthy tissues, can be processed into antigenic peptides in tumor cells and presented via major histocompatibility complex class I molecules, serving as novel tumor-associated antigens (20). ERV reactivation can also promote immunogenic cell death and remodel the tumor immune microenvironment, collectively simulating viral infection and inducing a viral mimicry effect.

Immune checkpoint inhibitors (ICIs) constitute a class of therapeutics designed to restore and amplify the body’s intrinsic T cell-mediated antitumor immunity by targeting and blocking immunosuppressive signaling pathways (21). The fundamental mechanism of ICIs centers on releasing effector T cells from inhibitory signals imposed by the tumor microenvironment, thereby revitalizing the immune system’s capacity to recognize and eliminate malignant cells. Current primary clinical targets include programmed death protein−1 (PD−1) and its ligand PD−L1, as well as cytotoxic T lymphocyte-associated protein−4 (CTLA−4) (22).

PD−1 functions as an inhibitory checkpoint receptor constitutively expressed on activated immune cells, while PD−L1 is often inducibly expressed on antigen−presenting cells and tumor cells. Tumor−upregulated PD−L1 engages PD−1 on T cells, transmitting inhibitory signals that lead to T cell proliferative arrest, increased apoptosis, and functional exhaustion (23–25). In contrast, CTLA−4 primarily regulates early T cell activation. Within secondary lymphoid organs, CTLA−4 competes with the costimulatory receptor CD28 for binding to B7 molecules on antigen−presenting cells, thereby dampening initial T cell priming. Tumors can further recruit regulatory T cells (Tregs) that express high levels of CTLA−4, amplifying local immunosuppression and attenuating antitumor immunity (26, 27).

ICIs have emerged as a major breakthrough in cancer therapy, achieving FDA approval for more than 20 cancer types and improving both patient survival and objective response rates (21, 28). However, only a subset of patients benefit, and resistance remains a pervasive challenge, particularly in monotherapy regimens. Current evidence indicates that defects in antigen presentation, impaired Interferon-γ(IFN-γ) signaling, and the immunosuppressive tumor microenvironment collectively underlie ICI resistance (29–36). Within this context, the viral mimicry effect induced by ERV reactivation offers a novel biological perspective and may serve as a key link between epigenetic abnormalities and anti-tumor immune responses. Moreover, ERV activation has the potential to function as a predictive biomarker for ICI efficacy.

In this review, we systematically summarize the latest research advances in the field of ERVs and ICI therapy. First, we elucidate how ERV reactivation triggers antitumor immune responses through viral mimicry and reshapes the tumor immune microenvironment. Subsequently, we explore the potential value of ERV expression profiles as novel biomarkers for predicting the efficacy of ICIs. Finally, we elaborate on therapeutic strategies aimed at targeting ERVs to sensitize tumors to ICI treatment, addressing both current challenges and future translational directions.

## 2 Endogenous retroviruses in cancer

### 2.1 Mechanisms of ERV reactivation in tumors

**Figure 2:**
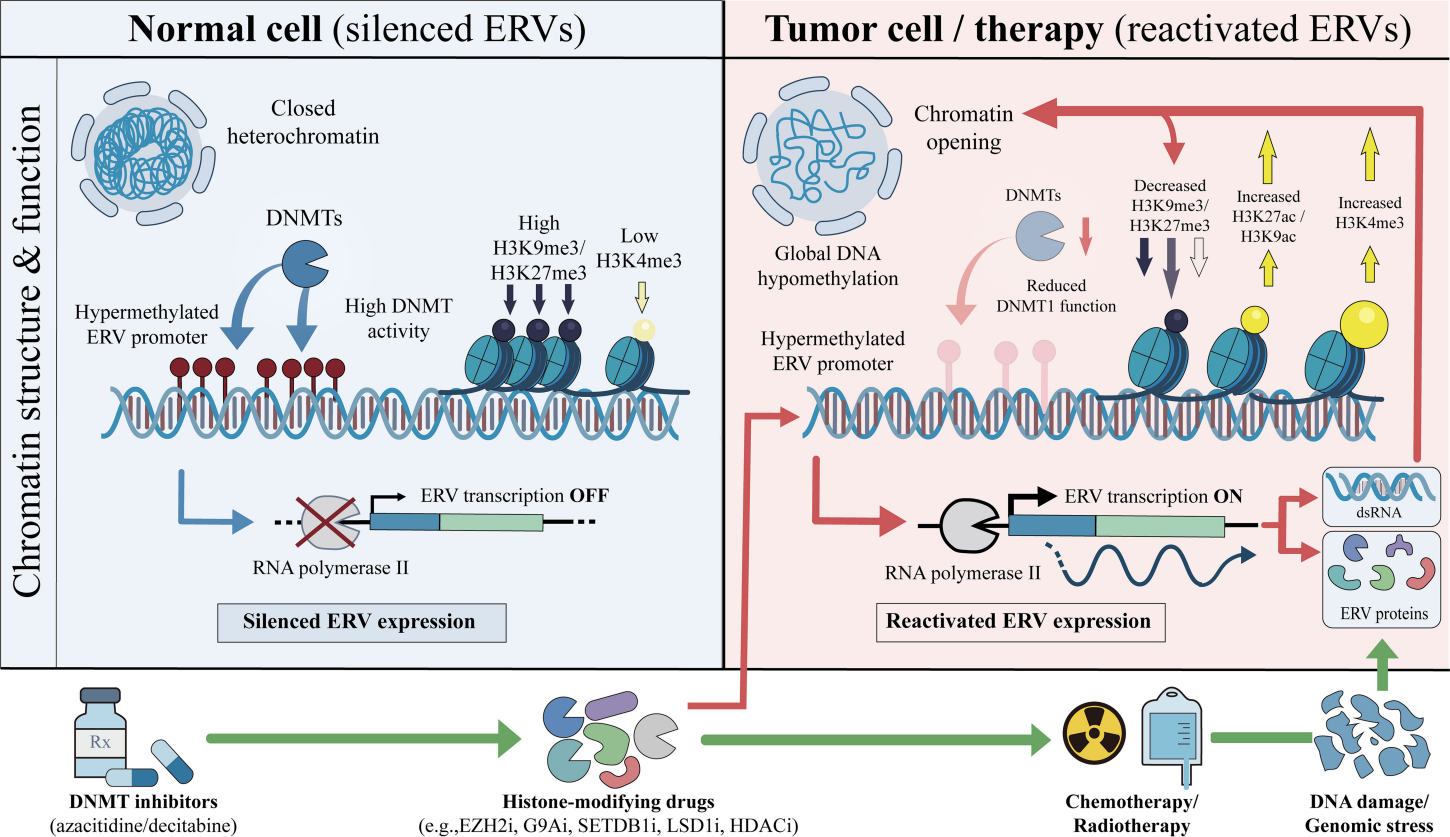
Major mechanisms underlying endogenous retrovirus (ERV) reactivation in cancer. Under normal conditions (left), ERV loci are maintained in a transcriptionally silent state by a compact heterochromatin configuration, high DNA methyltransferase (DNMT) activity, DNA hypermethylation at ERV promoters, and enrichment of repressive histone modifications, including H3K9me3 and H3K27me3, together with low levels of activating marks such as H3K4me3. These features limit RNA polymerase II recruitment and maintain ERV transcription in the OFF state. In tumor cells or under therapeutic stress (right), global epigenetic dysregulation promotes ERV derepression through chromatin opening, global DNA hypomethylation, reduced DNMT function (including impaired DNMT1 activity), loss of repressive histone marks, and acquisition of transcriptionally permissive chromatin states marked by increased H3K27ac, H3K9ac, and H3K4me3. These changes enable RNA polymerase II access and switch ERV transcription ON. Reactivated ERVs can produce viral RNA species, including double-stranded RNA (dsRNA), and in some settings also generate ERV-derived proteins or peptides, which may engage innate immune sensing pathways and contribute to antitumor immunity through viral mimicry. The lower panel highlights representative anticancer treatments that can enhance ERV expression, including DNMT inhibitors (e.g., azacitidine and decitabine), histone-modifying agents (e.g., EZH2, G9a, SETDB1, LSD1, or HDAC inhibitors), and chemotherapy or radiotherapy, as well as DNA damage/genomic stress. Despite diverse upstream triggers, these pathways converge on epigenetic ERV derepression and downstream induction of viral mimicry.

During cancer progression, intrinsic epigenetic dysregulation and therapeutic interventions can induce derepression and aberrant activation of ERVs. Key mechanisms include DNA demethylation and chromatin remodeling: promoter hypomethylation and changes in histone marks (e.g., decreased H3K9me3 and increased H3K4me3) open heterochromatin, allowing ERV transcription (17, 18, 37–44). Conventional chemotherapy, radiotherapy, and DNA Methyltransferase (DNMT) inhibitors (e.g., azacitidine, decitabine) can also trigger ERV expression, with DNMT inhibitors’ potent derepression contributing to their immunomodulatory effects (3, 45–48). Overall, diverse upstream events converge on epigenetic derepression to activate ERVs (**Figure 2**; **Table 1**).

**Table 1:** Major mechanisms of ERV reactivation in tumors and their clinical implications.

Mechanism	Key events	Effect on ERVs	Clinical relevance	References
DNA demethylation (endogenous)	DNMT activity dysregulation	ERV desilencing; aberrant transcription	Epigenetic reprogramming of cancer cells	(18, 40, 41)
DNA demethylation (therapeutic)	DNMT inhibitors	Strong ERV activation; dsRNA production	Enhancement of tumor immunogenicity	(38, 39, 41, 45, 48)
Histone modification	H3K9 reduction,H3K4 increase	Increased ERV transcriptional activity	Potential therapeutic target	(37, 42–44)
Radiotherapy	Ionizing radiation	ERV expression upregulated	Activation of anti-tumor immune responses	(47)

Regarding immunotherapy, specifically ICI therapy, current evidence does not sufficiently support the notion that ICIs directly regulate endogenous retrovirus (ERV) reactivation. Instead, the ERV upregulation observed during ICI treatment is predominantly attributable to (1) pre-existing, tumor-intrinsic epigenetic dysregulation and (2) concurrent or sequential ERV-inducing interventions, most notably epigenetic therapies (e.g., DNMT or HDAC inhibitors) and, in select contexts, cytotoxic regimens that disrupt chromatin architecture. Within this framework, ICIs primarily exploit a permissive ERV state by alleviating T cell suppression, whereas pharmacologic ERV induction serves as an immune-priming strategy that amplifies viral mimicry and type I interferon signaling, thereby sensitizing tumors to ICI therapy.

### 2.2 The ERV expression landscape across cancer types

In current research, ERVs have been found to be highly expressed in various tumors, with their expression patterns demonstrating significant specificity and heterogeneity. Specifically, tumors from different tissue origins overexpress distinct ERV families, and ERV expression profiles can also differ across subtypes within the same cancer. In renal cell carcinoma, several ERV families show elevated transcription compared with normal kidney tissue, and ERV-K expression is most pronounced in clear cell renal cell carcinoma (ccRCC) (49, 50). In colorectal cancer, aberrant activation of multiple ERV families (e.g., ERV1 and ERVL-MaLR) has been reported (51), and autosomal ERV expression profiles can stratify patients into four subtypes with distinct immune microenvironments (52). In hematological malignancies, HERV expression is likewise heterogeneous, distinguishing acute myelocytic leukemia (AML) differentiation subtypes and Hodgkin lymphoma from other subtypes largely characterized by HERV-F downregulation (**Table 2**) (53).

**Table 2:** ERV expression landscape across cancer types.

Cancer type	ERV family	Expression pattern/feature	References
Renal cell carcinoma	Multiple ERV families	Upregulation of expression in tumor tissue	(49)
Clear cell RCC	ERV-K	High expression of ERV-K env	(50)
Colorectal cancer	ERV1, ERVL-MaLR, etc.	Abnormal Activation of Multiple ERV Families	(51, 52)
Acute myeloid leukemia	HERV-E, HERV-K	Distinct expression profiles across AML subtypes	(53)
		Downregulation of HERV-F in all leukemia subtypes	
Lymphoma	HERV-F, others	Differential expression concentrated in Hodgkin lymphoma	(53)
		Downregulation of HERV-F in non-Hodgkin lymphoma	

### 2.3 ERVs as emerging biomarkers for diagnosis and prognosis

Tumor-specific and heterogeneous ERV expression patterns suggest that ERVs may serve as prognostic biomarkers in cancer. Expression of specific ERV families or loci has been associated with patient outcomes, including survival, across multiple studies (40, 51, 54–56).

In colorectal cancer, the LTR7 element from the HERV-H family has been reported to drive cancer-specific expression of SLCO1B3. Its activity is associated with progression-free survival (40). Additionally, aberrant expression of the MER61C-LTR element (ERV1 family) has been linked to chemotherapeutic sensitivity in colorectal cancer (51).

Beyond single elements, ERV expression profiling can stratify patient cohorts. For example, Bhagwate et al. classified colorectal cancer into four subtypes based on autosomal ERV expression. These subtypes differed in immunogenic signature scores, and differentially upregulated genes were enriched in antimicrobial immune response pathways (52). Together, these findings suggest that the association between ERVs and cancer prognosis is not merely an independent correlation, but may involve specific immune states driven by ERV activation.

## 3 ERV activation and anti-tumor immunity

ERV-derived transcripts can act as molecular triggers of anti-tumor immunity. In this context, ERV-derived dsRNA is a key mediator of viral mimicry (57–59).

Endogenous dsRNA mainly arises via two routes: (i) transcription of ERV long terminal repeats containing inverted repeats that fold into stem–loop structures; and (ii) annealing of complementary single-stranded RNAs produced from different ERV loci or by bidirectional transcription of the same element (19, 42, 60–62). Under physiological conditions, these dsRNA are edited by RNA-specific adenosine deaminase 1(ADAR1) through A-to-I conversion, which disrupts dsRNA structures, promotes their degradation, and thereby preserves immune tolerance (42, 61, 62).

When external factors induce epigenetic reprogramming, leading to the derepressed expression of ERVs, dsRNA is consequently produced in large quantities. Although adenosine deaminase 1 levels vary across tumors, the rate of dsRNA generation driven by ERV reactivation is often sufficient to exceed the editing and degradative capacity threshold of adenosine deaminase 1 (42, 61, 63). Subsequently, accumulated dsRNA is sensed by pattern recognition receptors (PRRs), triggering downstream innate immune signaling.

Sensing of ERV-derived dsRNA by host pattern recognition receptors directly links ERV activation to innate immune responses. This recognition process is primarily orchestrated by two classes of receptors: the cytoplasmic RIG-I-like receptors (RLRs), namely retinoic acid-inducible gene I (RIG-I) and melanoma differentiation-associated protein 5 (MDA5), and the endosomally localized Toll-like receptor 3 (TLR3) (64, 65).

Although both melanoma differentiation-associated protein 5 and retinoic acid-inducible gene I are RIG-I-like receptors, they differ in the dsRNA features they preferentially recognize. Retinoic acid-inducible gene I preferentially binds short dsRNA (typically <~1 kb), especially those bearing a 5′-triphosphate (5′ppp) end, which is common in certain viral replication intermediates or short viral RNA fragments. In contrast, melanoma differentiation-associated protein 5 preferentially recognizes long dsRNA (>~1 kb) that generally lacks 5′ppp ends, a profile consistent with ERV-derived dsRNA (19, 60, 66–68).

Ligand binding induces conformational changes in retinoic acid-inducible gene I or melanoma differentiation-associated protein 5 that expose their caspase recruitment domains. The caspase recruitment domains then engage the caspase recruitment domain of mitochondrial antiviral signaling protein (MAVS), promoting mitochondrial antiviral signaling protein oligomerization. This drives assembly of a downstream signaling complex on the outer mitochondrial membrane (69–71).

Like melanoma differentiation-associated protein 5, Toll-like receptor 3 recognizes long dsRNA, but it does so within endosomes. As Toll-like receptor 3 is localized to endosomal membranes, it cannot directly access cytosolic dsRNA. Cytosolic dsRNA must therefore be delivered into the endosomal lumen via endocytosis for Toll-like receptor 3 recognition. Following ligand binding, Toll-like receptor 3 undergoes homodimerization and a conformational change. This enables it to recruit the adaptor protein TIR-domain-containing adapter-inducing interferon-β (TRIF) via its intracellular Toll/interleukin-1 receptor (TIR) domain, thereby initiating downstream signaling (19, 59, 72). Notably, studies indicate that endogenous dsRNA triggers weaker activation of the Toll-like receptor 3 pathway than of the melanoma differentiation-associated protein 5 pathway. This difference may stem from Toll-like receptor 3’s endosomal localization and the additional steps required for ligand delivery (60).

Ultimately, the TLR3-TRIF and RIG-I/MDA5-MAVS pathways converge on activation of interferon regulatory factor 3/7 (IRF3/7) and nuclear factor kappa B (NF-κB). Both TIR-domain-containing adapter-inducing interferon-β and mitochondrial antiviral signaling protein, upon activation, recruit and activate the TANK-binding kinase 1 (TBK1) and the IκB kinase (IKK) complex. TANK-binding kinase 1 and IκB kinase ϵ subsequently phosphorylate interferon regulatory factor 3/7, leading to their dimerization and translocation into the nucleus. Concurrently, the IκB kinase complex phosphorylates IκBα, triggering its degradation and thereby releasing nuclear factor kappa B for nuclear import.

In the nucleus, interferon regulatory factor 3/7 and nuclear factor kappa B recruit coactivators such as CREB-binding protein (CBP) and p300. Together, they engage cis-regulatory elements in type I interferon promoters (e.g., the IFN-β enhanceosome and the interferon-sensitive response elements on IFN-α promoters) to initiate transcription. Newly synthesized IFN-α/β are secreted and signal in autocrine and paracrine manners via the interferon-α/β receptor (IFNAR) on the producing and neighboring cells (60, 63, 73, 74).

**Figure 3:**
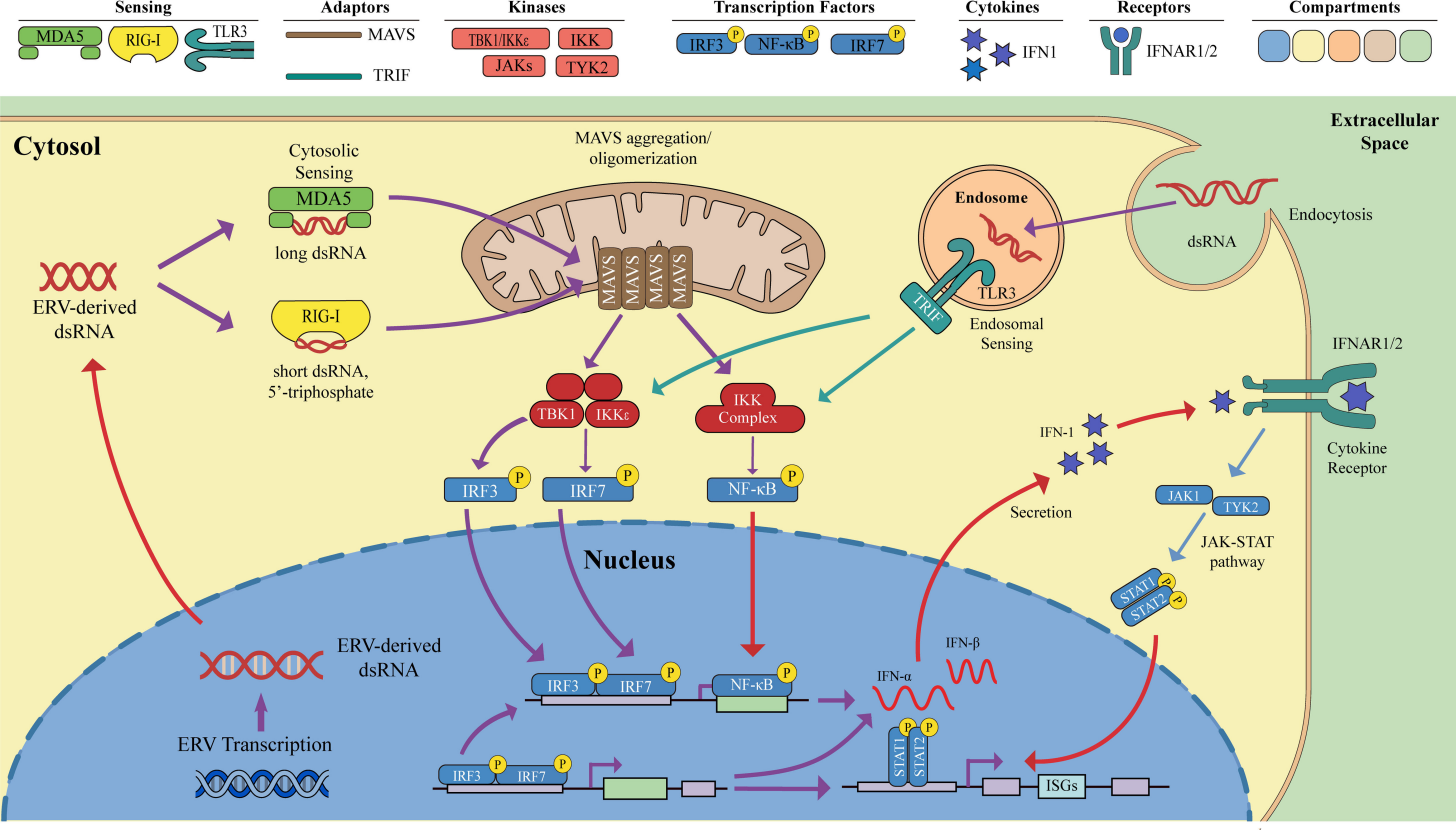
ERV-derived double-stranded RNA (dsRNA) sensing pathways that drive viral mimicry and type I interferon signaling. Reactivated endogenous retroviruses (ERVs) generate RNA transcripts that can form dsRNA, thereby mimicking viral infection and activating innate immune pathways. In the cytosol, ERV-derived dsRNA is sensed by the RIG-I-like receptors MDA5 and RIG-I, which recognize distinct RNA ligands: MDA5 preferentially detects long dsRNA, whereas RIG-I recognizes short dsRNA bearing 5′-triphosphate ends. Both receptors signal through mitochondrial antiviral-signaling protein (MAVS) on the outer mitochondrial membrane, where MAVS oligomerization promotes downstream kinase activation. In parallel, dsRNA that enters the endosomal compartment through endocytosis can be detected by Toll-like receptor 3 (TLR3), which signals via TIR-domain-containing adapter-inducing interferon-β (TRIF). These cytosolic and endosomal sensing pathways converge on TBK1, IKKϵ, and the IKK complex, resulting in phosphorylation and activation of the transcription factors IRF3, IRF7, and NF-κB. Activated IRF3/IRF7 and NF-κB translocate to the nucleus, where they induce transcription of type I interferons, including IFN-α and IFN-β. Secreted type I interferons then act in an autocrine and paracrine manner through IFNAR1/2 on the cell surface, triggering the JAK1/TYK2–STAT1/STAT2 signaling cascade and promoting the expression of interferon-stimulated genes (ISGs). Together, these pathways illustrate how ERV reactivation elicits a viral mimicry program that amplifies innate immune signaling and supports downstream antitumor immune responses.

Type I interferons (IFN-I) are key cytokines that shape antitumor immunity within the tumor microenvironment (TME). Through binding to the broadly expressed interferon-α/β receptor 1/2 receptor complex, IFN-I activate Janus kinase–signal transducer and activator of transcription (JAK-STAT) signaling, thereby inducing the broad expression of hundreds of interferon-stimulated genes (ISGs) (**Figure 3**) (75–77). Functionally, interferon-stimulated genes enhance antigen processing and presentation, promote immune-cell recruitment, modulate cell apoptosis and proliferation, and reversing immunosuppression. Together, these effects render the TME more permissive to immune surveillance and tumor elimination (42, 63).

Specifically, ISG-mediated immune reprogramming primarily involves the following key changes: (1) Upregulates MHC-I and components of the antigen-processing machinery in tumor cells, enhancing antigen presentation to CD8^+^ T cells; (2) Induces chemokines, which specifically recruit CXCR3-expressing effector CD8^+^ T cells, Th1 cells, and natural killer cells to infiltrate the tumor site; (3) Promotes dendritic cell (DC) maturation and activation. It also reduces infiltration of regulatory T cells (Tregs) and myeloid-derived suppressor cells (MDSCs) and drives macrophages polarizing toward a pro-inflammatory, antigen-presenting M1 phenotype; (4) Facilitates tumor vascular normalization, improving immune-cell extravasation and intratumoral distribution; (5) Directly induces tumor cell apoptosis and inhibition of tumor cell proliferation through cytotoxic and pro-apoptotic genes among interferon-stimulated genes (76, 78–81).

Taken together, ERV reactivation generates dsRNA that is sensed by pattern-recognition receptors, activating a type I interferon program and broadly inducing interferon-stimulated genes that remodel the TME. As a result, the TME shows increased antigen presentation, greater infiltration of effector immune cells, and attenuated immunosuppressive constraints. These changes can further potentiate pre-existing immune-hot tumors and may also promote the transition of immune-cold tumors toward an immune-inflamed state, thereby increasing responsiveness to ICIs.

As noted earlier, ICI efficacy requires an immunologically active TME with effector-cell infiltration and effective antigen recognition, whereas these conditions are often limited by the immunosuppressive state prevalent in many tumors. ERV-driven remodeling of the TME helps create a permissive context for ICI activity. First, ISG-induced chemokines recruit effector immune cells into tumors, providing the cellular substrate required for ICIs to act. Secondly, enhanced immunogenicity contributes to a more robust anti-tumor immune response by providing stronger antigenic signals for immune cell activation. Lastly, the inflammatory TME shaped by ERV activation can drive a feedback upregulation of PD-L1 through ISG-mediated mechanisms, thereby providing a well-defined intervention target for ICIs.

## 4 Clinical evidence

### 4.1 ERV expression as a biomarker for ICI response

Given that ERV activation can remodel the tumor immune microenvironment, intratumoral ERV expression has been proposed as a biomarker to predict responses to ICIs. Bioinformatic studies have revealed that across multiple tumor types, a high transcriptional level of either overall or specific ERV families within tumor tissue is significantly associated with improved patient survival following ICI therapy (82–84).

For example, in clear cell renal cell carcinoma, Smith et al. analyzed TCGA RNA-seq data and quantified the expression of 3,173 full-length ERV loci. They found that the expression of multiple ERV subtypes was independently associated with patient survival, consistent with the immunoregulatory pathways described above. They further reported that PD-1 inhibitor responders with clear cell renal cell carcinoma exhibited higher expression of tumor-specific HERVs (e.g., HERV-4700). These HERV-derived epitopes can bind human leukocyte antigen (HLA) molecules, supporting antigen presentation and T-cell activation (20).

Similarly, in hepatocellular carcinoma (HCC), Wen et al. reported the correlation between ERVK3–1 expression and ICI efficacy as well as patient prognosis through statistical analysis of the liver hepatocellular carcinoma cohort in TCGA and validation via *in vitro* cell experiments. High ERVK3–1 expression in hepatocellular carcinoma tumors was associated with higher pathological grade (G3–G4), advanced clinical stage (III–IV), and elevated alpha-fetoprotein levels (>400 ng/mL). Furthermore, it served as an independent prognostic factor for poor overall survival (OS) and disease-specific survival (DSS). Consistently, patients with lower ERVK3–1 expression had higher ICI response rates, whereas higher ERVK3–1 expression was associated with treatment resistance (85).

### 4.2 ERV-based stratification

Stratifying patients by ERV expression profiles may add a new layer to precision immunotherapy. Such stratification can predict responses to ICI monotherapy and help identify subgroups that may benefit from combination regimens (84). Currently, most ERV-based stratification models construct comprehensive scores by integrating the opposing prognostic effects of distinct ERVs, categorized as protective versus risk-associated types.

For example, Zhou et al. used univariable Cox and LASSO regression to select nine ERVs and construct an ERV-based risk score. Patients were then stratified into low- and high-risk groups. In the low-risk group, nivolumab was associated with better overall survival (OS), progression-free survival (PFS), and objective response rate (ORR) than everolimus. By contrast, outcomes did not differ significantly between nivolumab and everolimus in the high-risk group (86).

In metastatic clear cell renal cell carcinoma, Lu et al. identified two ERV loci with opposite clinical associations. E4421_chr17 was risk-associated, whereas E1659_chr4 was protective. They constructed four distinct subgroups based on the median expression levels of these two loci, which were further integrated into a three-tiered risk model (ERV3). In this model, low-risk patients had significantly longer PFS and OS than high-risk patients. ERV3 outperformed established transcriptomic signatures (Angio, Teff, and TIS) in predicting response to ICI therapy. Furthermore, combining ERV3 with the DNA methylation marker iMES further improved the prediction of resistance to ICI therapy (87).

At present, stratification models based on ERV expression require further validation. Early clinical evidence largely comes from retrospective analyzes of completed immunotherapy cohorts. For instance, one study in non-small cell lung cancer (NSCLC) measured ERV expression by RNA sequencing and found that high ERV expression was independently associated with a higher ORR to anti–PD-1/PD-L1 therapy. High ERV expression was also associated with longer PFS. Stratified analyzes further suggested that high ERV expression remained associated with benefit even in ICI-refractory molecular subsets (e.g., EGFR-mutant or ALK-rearranged tumors). Mechanistically, high ERV expression correlated with higher expression of type I interferon signaling genes and increased intratumoral CD8^+^ T-cell infiltration. These findings are consistent with a more inflamed tumor microenvironment and may help explain responses in subsets otherwise resistant to ICI therapy (84).

### 4.3 Clinical validation and prospective trials

Stronger clinical evidence is provided by prospectively designed combination-therapy trials. For example, in a phase II study focusing on relapsed/refractory NK/T-cell lymphoma, Huang et al. conducted integrated analyzes using patient samples, cell lines, and mouse models. They showed that DNA-demethylating agents demethylate ERV promoter regions and increase ERV transcription. ERV reactivation triggered downstream dsRNA-MAVS-type I interferon signaling. This process simultaneously promoted tumor infiltration by CD8^+^ T cells and alleviated their exhausted state, providing a mechanistic explanation for how induced ERV reactivation can reverse resistance to ICIs (88).

However, the success of this combination strategy is not universal, and its efficacy appears highly contingent upon achieving effective and sufficient DNA demethylation in the target tumors. A Phase II study in advanced solid tumors (the METADUR trial) provided robust counter-evidence in this regard.

METADUR tested whether CC−486 (oral azacitidine) could prime immunologically “cold” tumors for PD−L1 blockade with durvalumab. The enrolled tumor types included microsatellite-stable colorectal cancer, platinum-resistant ovarian cancer, and ER-positive/HER2-negative breast cancer. Although the combination regimen was safe and feasible, no objective clinical responses were observed. In-depth biomarker analysis revealed the core reason for this failure: neither under the high-dose, short-course regimen nor the low-dose, long-course regimen combined with vitamin C was significant global or genome-wide DNA demethylation detected in patients’ peripheral blood or paired tumor biopsy samples. Moreover, the research team directly interrogated transcriptomic alterations in repetitive elements within tumor specimens using RNA sequencing and similarly detected no ERV upregulation. Consequently, the anticipated viral mimicry effect and remodeling of the tumor microenvironment did not occur (89).

Moreover, certain clinical studies—although they did not directly measure ERV expression levels—still offer indirect evidence supporting ERV-mediated immune activation mechanisms, based on the treatment modalities employed and the observed immunophenotypic changes.

For example, a phase II neoadjuvant study in locally advanced HER2-negative breast cancer evaluated decitabine plus pembrolizumab and reported marked immune-microenvironment changes, including reduced myeloid-derived suppressor cells, increased tumor-infiltrating lymphocytes, and higher PD-L1 expression. These immunophenotypic shifts were associated with clinical benefit (90).

In the SCENT Ib/II study (Gao et al.), chidamide (an HDAC inhibitor) plus sintilimab was evaluated in relapsed/refractory NK/T-cell lymphoma. The regimen achieved an objective response rate of 59.5% and a complete response rate of 48.6%; the median duration of response was 25.3 months, with median progression-free survival and overall survival of 23.2 and 32.9 months, respectively. These outcomes exceeded the historical complete response rate of ~30% reported with prior PD-1 monotherapy, suggesting improved depth and durability of response with the combination (91).

In summary, evidence ranging from retrospective correlative analyzes to prospective interventional trials supports ERV-associated features as candidate predictive biomarkers. Concurrently, the pharmacological induction of ERV reactivation through agents such as epigenetic drugs has shown potential as a synergistic strategy to enhance the efficacy of ICI therapy (**Table 3**).

**Table 3:** Summary of clinical evidence for ERV-related applications across cancer types.

Application direction	Cancer type	ERV family/element	Clinical relevance	References
ICI efficacy prediction	Clear cell renal cell carcinoma	HERV-E, HERV-K, etc.	High ERV expression reflects good ICI response	(20)
	Hepatocellular carcinoma	ERVK3	The expression of ERV is associated with the efficacy of ICI	(85)
Risk stratification	Clear cell renal cell carcinoma	Multiple ERV families	Low risk score predicts favorable ICI response	(86)
	Clear cell renal cell carcinoma	ERVK, ERV1, ERVL-MaLR	Low risk group predicts better ICI efficacy	(87)
	Non-small cell lung cancer	ERV1	High expression associated with ICI benefit	(84)
Enhancing efficacy	NK/T-cell lymphoma	ERV1, ERV3, ERVK	The reversal of ICI resistance	(88)

## 5 Therapeutic implications

**Figure 4:**
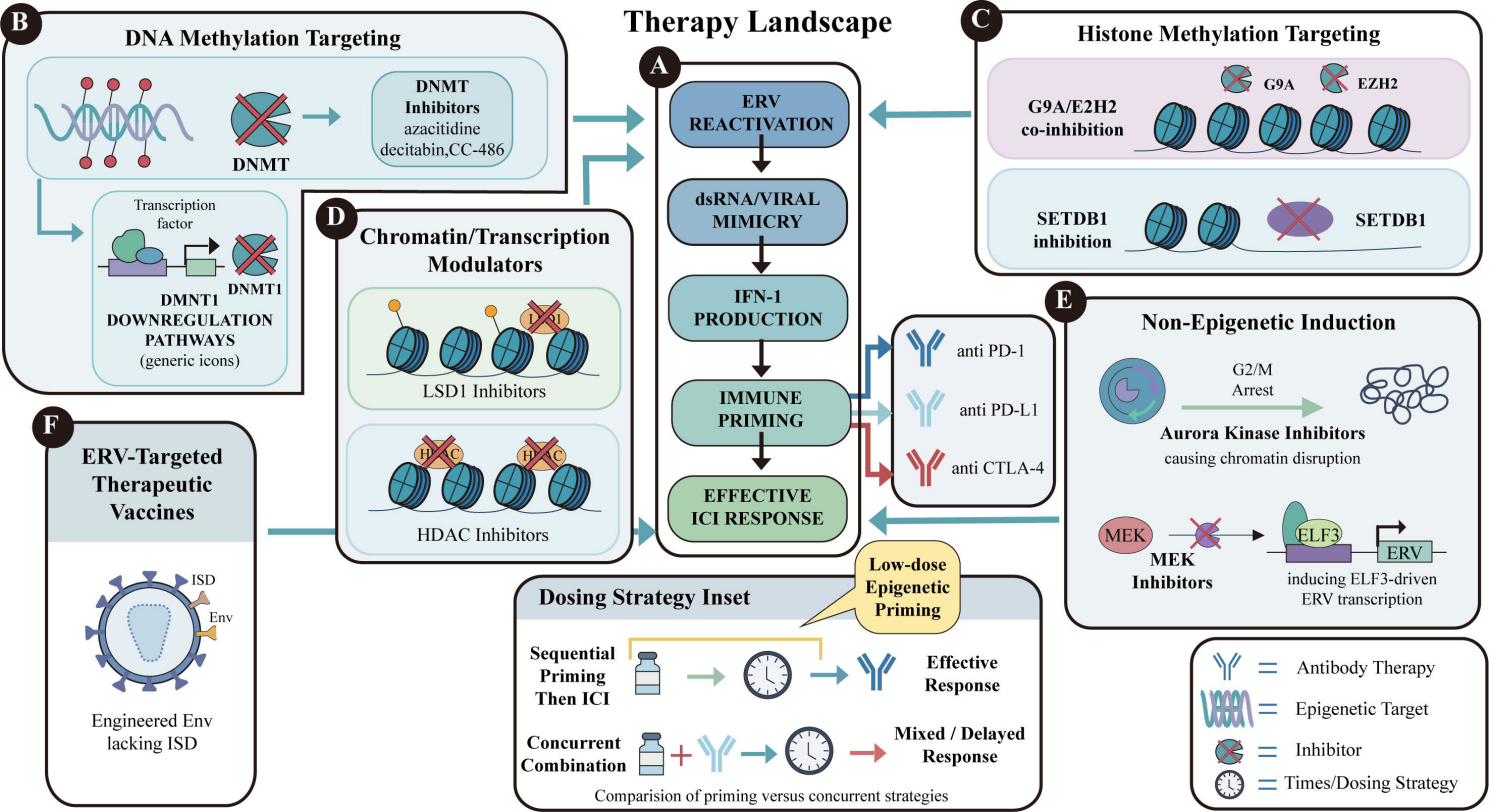
Therapeutic strategies to induce endogenous retrovirus (ERV) reactivation and exploit viral mimicry to enhance immune checkpoint blockade. **(A)** The central panel summarizes the proposed therapeutic framework: induction of ERV reactivation leads to accumulation of double-stranded RNA (dsRNA) and viral mimicry, followed by type I interferon (IFN-I) production, immune priming, and ultimately a more effective response to immune checkpoint inhibition (ICI). This rationale supports combination with antibodies targeting PD-1, PD-L1, or CTLA-4. **(B)** DNA methylation-targeting strategies include DNA methyltransferase (DNMT) inhibitors, such as azacitidine, decitabine, and CC-486, as well as pathways that reduce DNMT activity or expression, thereby relieving ERV silencing. **(C)** Histone methylation-targeting strategies include inhibition of repressive chromatin regulators such as G9a/EZH2 and SETDB1, which can promote chromatin relaxation and ERV transcription. **(D)** Chromatin and transcription modulators, including LSD1 inhibitors and histone deacetylase (HDAC) inhibitors, may further enhance ERV expression by reshaping chromatin accessibility and transcriptional permissiveness. **(E)** Non-epigenetic ERV-inducing approaches may also promote ERV activation indirectly. Examples shown here include aurora kinase inhibitors, which can induce G2/M arrest and chromatin disruption, and MEK inhibitors, which may increase ELF3-driven ERV transcription. **(F)** ERV-targeted therapeutic vaccines represent an additional strategy to amplify antitumor immunity by exploiting ERV-derived antigens; the schematic depicts an engineered Env immunogen lacking the immunosuppressive domain (ISD). The inset illustrates potential dosing strategies, highlighting the concept that low-dose epigenetic priming administered before ICI may improve therapeutic responses compared with concurrent administration in some settings. Together, these approaches position ERV induction as a bridge between epigenetic therapy and cancer immunotherapy.

Extensive preclinical studies have elucidated mechanisms underlying ERV induction and its potential synergy with ICI. Exploratory clinical studies have also reported associations between ERV expression and treatment response, but translation into clinical strategies remains at an early stage. Accordingly, the emphasis is shifting from mechanistic validation to developing clinically actionable translation strategies. ERVs are transcriptionally silenced in somatic cells by multiple epigenetic controls, including DNA methylation and repressive chromatin modifications (13). Therefore, current therapeutic strategies are largely built on reversing these barriers to reactivate ERVs and trigger a viral mimicry response (**Figure 4**; **Table 4**) (3).

**Table 4:** Summary of therapeutic strategies and targets for ERV reactivation.

Activation strategy	Target/mechanism	Activation method	References
DNA demethylation	DNMT1	DNMTi	(92)
		methyltestosterone	(93)
		CDK4/6 inhibitors	(94)
Targeting histone methyltransferases	G9A, EZH2	Dual-target inhibitors	(98)
		Combination target inhibitors	(99)
	SETDB1	CRISPR-Cas9 knockout	(100)
		Small molecule inhibitors	(101)
Targeting histone demethylases	KDM1A	Inhibitors	(60, 102)
	KDM3A	Gene knockout	(58)
		Inhibitors	(58)
Non-epigenetic strategies	Physical disruption of nuclear structure	Aurora kinase inhibitors	(107)
	Upregulate expression of transcription factor ELF3	MEK1/2 inhibitors	(108)

### 5.1 Targeting DNA methylation for ERV reactivation

DNA demethylation is among the best-characterized mechanisms and the most clinically advanced approaches for ERV activation. Conventional approaches primarily rely on nucleoside analog DNMT inhibitors such as decitabine, which induce genome-wide non-selective DNA demethylation through covalent trapping of DNMT1 (92).

Recent work has expanded DNA methylation-modulating strategies beyond direct inhibition of DNMT enzymatic activity. Several studies have shown that DNMT1, a key maintenance factor for ERV silencing, is dynamically regulated by upstream signaling at both the transcriptional and post-translational levels. Targeting these upstream pathways can reduce DNMT1 abundance or activity, thereby promoting DNA demethylation. For example, in AR-positive prostate cancer, methyltestosterone binds the androgen receptor and downregulates DNMT1 and DNMT3B, resulting in genome-wide hypomethylation (93). Concurrently, CDK4/6 inhibitors reduce DNMT1 protein levels by inhibiting E2F transcriptional activity, which subsequently relieves the silencing of ERV3–1 in specific infection models (94).

Although DNA demethylation has shown efficacy in preclinical models and advanced into multiple clinical trial phases, its intrinsic lack of selectivity remains a major limitation. This nonselectivity constrains its clinical deployment. Genome-wide demethylation may increase genomic instability and mutagenic risk, and it can confound attribution of clinical effects to beneficial ERV activation versus genotoxicity (95–97). Consequently, the modulation of histone methylation modifications, which enables more precise regulatory control, is emerging as a promising and translationally viable research direction.

### 5.2 Histone modifications in ERV reactivation

#### 5.2.1 Targeting histone methylation

Histone methylation–targeted strategies primarily act by remodeling ERV-associated repressive chromatin, which is maintained by deposition of H3K9me2/3 and H3K27me3 and by reduced levels of the activating mark H3K4me2.

Targeting histone methyltransferases is a central approach to modulating ERV chromatin repression. Co-inhibition of G9A (EHMT2) and EZH2—responsible for H3K9me2/3 and H3K27me3 deposition, respectively—reduces these repressive marks and derepresses ERV transcription. This effect has been achieved using the dual inhibitor HKMTI-1–005 or the combination of GSK126 (EZH2) and UNC0638 (G9A), leading to robust activation of type I interferon signaling (98, 99).

Targeting the H3K9me3 methyltransferase SETDB1 can also reverse H3K9me3-mediated silencing at ERV loci. This has been demonstrated by genetic disruption of the ATF7IP–SETDB1 complex (e.g., CRISPR–Cas9 knockout) or by small-molecule inhibition (e.g., SETDB1-TTDIN-1), resulting in ERV derepression and increased tumor antigen expression (100, 101).

An alternative approach is to inhibit histone demethylases, thereby increasing chromatin accessibility by preserving or enhancing activating histone marks. Pharmacologic inhibition of the H3K4 demethylase LSD1 (KDM1A) attenuates LSD1-associated chromatin compaction and restores ERV expression and interferon signaling in small cell lung cancer models (60, 102). In gastric cancer models, genetic ablation or pharmacologic inhibition of the H3K9 demethylase KDM3A increases H3K4me2 levels and activates ERV transcription (58).

#### 5.2.2 Targeting histone deacetylation

The therapeutic efficacy of combining histone deacetylase inhibitor(HDACi) with ICI has been validated in several studies. However, the relationship between this strategy and ERVs reactivation remains inadequately explored. These investigations have primarily focused on non-ERV-related mechanisms, such as remodeling of the immune microenvironment and direct regulation of PD-L1 transcription (103–105).

In a study of leiomyosarcoma cells, HDAC1/2/3 inhibitors were shown to significantly upregulate the expression of the ERV1 and ERVK families. Further mechanistic analysis revealed that this upregulation was closely associated with increased H3K27ac levels at the LTR12 region. However, no activation of the interferon response was observed in that study, and the investigators did not further evaluate the combinatorial efficacy of HDACi and ICI in the context of ERV reactivation (106).

In summary, the clinical feasibility of combining HDACi with ICI has been established. However, the strategy of using HDACi to sensitize tumors to immune checkpoint blockade via ERV reactivation remains theoretical and exploratory.

### 5.3 Non-epigenetic mechanisms for ERV reactivation

Beyond direct modulation of DNA or histone marks, cell-cycle perturbation and activation of specific transcriptional programs have also been shown to promote ERV reactivation. Accordingly, these non-epigenetic approaches may provide therapeutic alternatives for patients who are refractory to established epigenetic therapies.

#### 5.3.1 Induction via cell cycle and chromatin disruption

This pathway primarily involves cell-cycle disruption that mechanically perturbs nuclear architecture, thereby relieving ERV silencing. For example, Aurora kinase inhibitors induce G2–M arrest in tumor cells, resulting in aberrant chromatin condensation and disrupted nuclear-envelope integrity. These architectural changes can render previously inaccessible ERV loci permissive to transcription, generating dsRNA and activating innate immune signaling (107).

#### 5.3.2 Induction via transcriptional factor activation

This pathway promotes ERV-directed transcriptional programs through modulation of upstream signaling cascades. MEK1/2 inhibitors (e.g., trametinib) have been shown to rapidly induce expression of the transcription factor ELF3 without overtly altering the global epigenetic state. ELF3 then directly engages and activates selected ERV regulatory elements that are relatively weakly repressed. In concert with factors such as IRF1, ELF3 initiates ERV transcription and downstream interferon signaling (108).

### 5.4 Combination therapy approaches

Inducing viral mimicry is not, in itself, the ultimate therapeutic goal. Rather, its value lies in shaping an immunologic context that enhances responsiveness to ICIs. Accordingly, combining ERV-inducing strategies with ICIs has become a major focus of translational research. A key challenge is to optimize these regimens to maximize synergistic antitumor activity.

#### 5.4.1 The optimal window for immune activation

Mechanistically, ERV reactivation and ICI efficacy appear to follow a temporal sequence. ERV reactivation generates viral-mimicry signals that remodel the tumor immune microenvironment, thereby creating conditions in which ICIs can more effectively relieve T-cell suppression and elicit antitumor immunity. Because this immune-priming process likely requires a defined time window, sequential administration—ERV induction followed by ICI treatment—may be biologically plausible and, in principle, could outperform concurrent combination therapy.

However, available preclinical studies and early-phase clinical trials have not conclusively validated this putative advantage, and neither strategy has consistently demonstrated superiority. There are two primary reasons for this. First, ERV inducers differ markedly in their pharmacokinetic and pharmacodynamic properties, resulting in variable timing of immune priming. Second, the baseline state of the tumor microenvironment and its immune composition are highly heterogeneous among patients, which substantially influences the intensity and kinetics of the immune response. Consequently, in clinical practice, the efficacy of concurrent versus sequential regimens may converge, blurring the distinction between these approaches (58, 63, 88, 90, 108).

#### 5.4.2 The case for low-dose strategies

Dose optimization remains a critical challenge for clinical translation. Historically, clinical dosing of DNMTi has been guided primarily by cytotoxicity, with the goal of directly killing tumor cells. In contrast, ERV-driven immune sensitization may be achieved at exposures well below the cytotoxic threshold. Early-phase trials are evaluating low-dose DNMTis in combination with ICIs. Preliminary data suggest that low-dose cohorts preserve immunomodulatory activity while exhibiting a more favorable safety and tolerability profile (88, 90).

At present, further studies are needed to determine how preliminary evidence on the safety and activity of low-dose DNMTi can be translated into standardized clinical protocols. Critically, whether this low-dose immunomodulatory paradigm can be generalized to other, mechanistically distinct classes of ERV inducers remains a central challenge for future research.

#### 5.4.3 ERV-targeted therapeutic vaccines

Therapeutic vaccines targeting ERV-encoded proteins as tumor antigens represent a promising strategy for use in combination with ICI therapy. A key advantage of this approach is its capacity to elicit ERV-specific effector T cells, which can synergize with ICIs to amplify antitumor immunity.

Current ERV vaccine design focuses on deleting or mutating the immunosuppressive domain (ISD) to attenuate immunosuppressive activity and enhance antigen immunogenicity. ERV envelope proteins, particularly those in the transmembrane subunit, often contain an ISD analogous to that found in the Env proteins of exogenous retroviruses (109). The function of the ISD was first elucidated through studies of the conserved retroviral Env peptide CKS17 (110). This peptide exhibits high similarity to conserved regions in the envelope proteins of retroviruses such as MuLV (110, 111). It has been shown to suppress lymphocyte proliferation, natural killer cell activity, and cytokine production. Subsequent studies demonstrated that the ISD is a critical functional domain mediating immune evasion by retroviruses *in vivo*. Furthermore, multiple ERV-derived Env proteins retain ISD-like sequences and immunomodulatory activity (109, 112).

Daradoumis et al. used targeted mutagenesis of the ISD in the murine endogenous retrovirus (MelARV) Env protein, thereby abrogating its immunosuppressive activity. The optimized vaccine induced a substantially higher frequency of MelARV-specific CD8+ T-cell responses than the control group. In combination with an anti–PD-1 antibody, the vaccine achieved tumor clearance in 80% of animals in a colorectal cancer model. It also conferred partial protective efficacy across additional cancer types (113).

Furthermore, Maldonado and colleagues focused on the ERV envelope protein ERVMER34-1, which is highly expressed in numerous cancers but shows low expression in normal tissues. By deleting a highly conserved segment encompassing the ISD, they abrogated the protein’s immunosuppressive activity and reduced off-target toxicity. In mice bearing established tumors (~300 mm³ at treatment initiation), a single dose of the vaccine combined with an anti–PD-L1 antibody achieved tumor clearance in 89% of animals. The authors additionally added the IL-15 superagonist N-803 to create a triplet regimen. This combination showed enhanced immunostimulatory activity and antitumor efficacy in an immune-cold tumor model (114).

### 5.5 Challenges and safety considerations

Although ERV activation has emerged as a promising strategy to potentiate immune checkpoint blockade, its clinical translation remains constrained by several key challenges. First, the association between ERV activation and ICI benefit is supported mainly by retrospective analyzes and small, non-randomized studies, with limited external and prospective validation. Second, ERV detection and quantification lack methodological standardization across signatures, cutoffs, and platforms, reducing cross-study comparability. Finally, the safety and toxicity boundaries of ERV activation remain insufficiently defined. Systemic ERV activation may compromise immune self-tolerance and provoke aberrant inflammation or autoimmune responses. Data from ongoing combination-therapy trials suggest that rates of severe immune-related adverse events are not substantially higher than those historically reported with ICI monotherapy. However, these comparisons are limited by small sample sizes and short follow-up. Accordingly, long-term safety remains uncertain, and comprehensive safety data for emerging ERV-modulating strategies are largely unavailable. Defining a clinically actionable therapeutic window for ERV-modulating strategies is therefore essential.

## 6 Future directions

As a pivotal link between epigenetic dysregulation and antitumor immunity, ERVs offer new conceptual frameworks and therapeutic targets to enhance the efficacy of ICIs. To translate mechanistic insights into clinical benefit, future work should focus on several priorities that address current bottlenecks and accelerate progress in this field.

First, the heterogeneity in current ERV detection methodologies presents a significant hindrance to comparing research outcomes and enabling clinical translation. A standardized, rigorously benchmarked analytical framework that is broadly accepted by the field is needed. Using high-quality reference genomes and curated databases, it will be essential to define a consensus, high-confidence catalog of immunogenicity-associated ERV loci. Subsequently, integrated biomarkers that surpass simple expression metrics—such as a scoring system combining ERV transcript abundance, chromatin accessibility, and downstream ISG expression—should be developed and validated. In parallel, these biomarkers should be implemented in clinically deployable assays compatible with routine clinical specimens (115).

Second, the clinical value of ERV biomarkers requires rigorous validation in large-scale, prospective, multi-center studies or randomized controlled trials of ICI therapy. These studies should aim to determine their independent prognostic value for key endpoints (e.g., ORR, PFS, OS) and, crucially, evaluate their comparative advantage over established biomarkers in head-to-head analyzes. Furthermore, systematic investigation of biomarker performance across diverse contexts is imperative. Such efforts are key to defining their optimal clinical utility and informing patient selection strategies.

Third, a deeper mechanistic understanding requires elucidating the spatial biology of ERVs within the tumor microenvironment using advanced technologies. Single-cell and spatial transcriptomics can precisely delineate the cellular origins (e.g., malignant cells or specific immune subsets) responsible for ERV expression. Employing techniques such as multiplex immunofluorescence or *in situ* sequencing enables the spatial visualization of associations between ERV-high regions and CD8+ T cell infiltration, tertiary lymphoid structure formation, and the expression of immune checkpoint molecules. Collectively, these approaches will clarify how ERVs locally sculpt an immune-hot niche and help distinguish protective from pathological ERV activation states (83, 116–119).

Therapeutic translation, informed by a comprehensive mechanistic understanding of ERVs, aims to develop and evaluate combination strategies that safely induce ERV expression. In addition to optimizing the dosing regimens of existing epigenetic drugs, greater emphasis should be placed on exploring the clinical application of precision epigenetic tools, such as epigenome editing technologies and selective small-molecule compounds.

Finally, the clinical translation of any novel therapeutic strategy requires a foundation of comprehensive safety evaluation. Robust, long-term monitoring frameworks should be integrated into future clinical trials to assess potential autoimmune risks and to systematically investigate mechanisms of resistance. For instance, tumor cells may evade ERV-mediated immune recognition by upregulating RNA-editing enzymes such as adenosine deaminase 1 or by inhibiting downstream signaling pathways. Elucidating these escape mechanisms will provide a rationale for developing sequential or combination approaches to overcome resistance (42, 120).

## 7 Conclusion

ERV reactivation in cancer can generate a viral mimicry state that functionally resembles acute viral infection. Epigenetic derepression enables abundant ERV transcription, producing endogenous dsRNA that is sensed by pattern-recognition receptors such as MDA5/RIG-I and, to a lesser extent, TLR3. These pathways converge on IRF3/7 and NF-κB to induce type I interferons and interferon-stimulated genes, thereby enhancing antigen presentation, promoting dendritic cell maturation, recruiting cytotoxic lymphocytes through CXCL9/10/11, and partially dismantling immunosuppressive components of the tumor microenvironment. In parallel, ERV-encoded proteins can serve as tumor-associated antigens presented by MHC-I, further increasing immune visibility. Collectively, ERV-driven innate activation and antigenicity can convert immune-cold tumors toward a more inflamed, ICI-permissive state.

Clinically, ERV expression patterns demonstrate prognostic and predictive value for therapeutic decision-making in the context of immune checkpoint inhibition. Specifically, ERV-derived signatures can help stratify patients according to their likelihood of benefit from or resistance to immune checkpoint blockade, complement established biomarkers such as PD-L1 and tumor mutational burden, and provide a rationale for combination therapies designed to harness ERV-induced immune activation for improved outcomes.

Looking forward, the field should prioritize standardizing ERV measurement and signature definitions, validating ERV-based biomarkers in well-designed prospective studies, and developing safer, more precise approaches to therapeutically modulate ERVs. Addressing heterogeneity, optimizing clinical implementation, and clarifying long-term safety and resistance mechanisms will be essential to translate ERV biology into routine immunotherapy practice.

## Glossary

ERV: Endogenous retrovirus
HERV: Human endogenous retrovirus
dsRNA: Double-stranded RNA
ICI: Immune checkpoint inhibitor
PD−1: Programmed death protein−1
PD-L1: Programmed death ligand 1
CTLA−4: Cytotoxic T lymphocyte-associated protein−4
DNMT: DNA methyltransferase
RIG-I: Retinoic acid-inducible gene I
MDA5: Melanoma differentiation-associated protein 5
TLR3: Toll-like receptor 3
MAVS: Mitochondrial antiviral signaling protein
IRF3/7: Interferon regulatory factor 3/7
NF-κB: Nuclear factor kappa B
IKK: IκB kinase
IFN-I: Type I interferons
TME: tumor microenvironment
ISG: Interferon-stimulated gene
OS: Overall survival
PFS: Progression-free survival
ORR: Objective response rate
HDACi: Histone deacetylase inhibitor
ISD: Immunosuppressive domain.
